# Ecological Modeling of *Aedes aegypti* (L.) Pupal Production in Rural Kamphaeng Phet, Thailand

**DOI:** 10.1371/journal.pntd.0000940

**Published:** 2011-01-18

**Authors:** Jared Aldstadt, Constantianus J. M. Koenraadt, Thanyalak Fansiri, Udom Kijchalao, Jason Richardson, James W. Jones, Thomas W. Scott

**Affiliations:** 1 Department of Geography, University at Buffalo, Buffalo, New York, United States of America; 2 Laboratory of Entomology, Wageningen University, Wageningen, The Netherlands; 3 Department of Entomology, Armed Forces Research Institute of Medical Sciences, Bangkok, Thailand; 4 Division of Entomology, Walter Reed Army Institute of Research, Silver Spring, Maryland, United States of America; 5 Department of Entomology, University of California, Davis, Davis, California, United States of America; 6 Fogarty International Center, National Institutes of Health, Bethesda, Maryland, United States of America; Centers for Disease Control and Prevention, United States of America

## Abstract

**Background:**

*Aedes aegypti* (L.) is the primary vector of dengue, the most important arboviral infection globally. Until an effective vaccine is licensed and rigorously administered, *Ae. aegypti* control remains the principal tool in preventing and curtailing dengue transmission. Accurate predictions of vector populations are required to assess control methods and develop effective population reduction strategies. *Ae. aegypti* develops primarily in artificial water holding containers. Release recapture studies indicate that most adult *Ae. aegypti* do not disperse over long distances. We expect, therefore, that containers in an area of high development site density are more likely to be oviposition sites and to be more frequently used as oviposition sites than containers that are relatively isolated from other development sites. After accounting for individual container characteristics, containers more frequently used as oviposition sites are likely to produce adult mosquitoes consistently and at a higher rate. To this point, most studies of *Ae. aegypti* populations ignore the spatial density of larval development sites.

**Methodology:**

Pupal surveys were carried out from 2004 to 2007 in rural Kamphaeng Phet, Thailand. In total, 84,840 samples of water holding containers were used to estimate model parameters. Regression modeling was used to assess the effect of larval development site density, access to piped water, and seasonal variation on container productivity. A varying-coefficients model was employed to account for the large differences in productivity between container types. A two-part modeling structure, called a hurdle model, accounts for the large number of zeroes and overdispersion present in pupal population counts.

**Findings:**

The number of suitable larval development sites and their density in the environment were the primary determinants of the distribution and abundance of *Ae. aegypti* pupae. The productivity of most container types increased significantly as habitat density increased. An ecological approach, accounting for development site density, is appropriate for predicting *Ae. aegypti* population levels and developing efficient vector control programs.

## Author Summary

Dengue infection is the leading cause of arbovirus illness worldwide with an estimated 2.5 billion people at risk. The primary dengue vector, *Ae. aegypti*, develops mainly in artificial containers in and around human dwellings. Often a small number of container types are responsible for a large proportion of adult mosquitoes in a region. To this point, most studies of *Ae. aegypti* production have failed to consider the spatial arrangement of development sites. For this investigation, mosquito populations and development sites were sampled in a spatially exhaustive manner over a four year time period in rural Kamphaeng Phet, Thailand. The data indicate that not only are some container types more productive than others, but that the local density of development sites has a large effect on the productivity of individual containers. Specifically, containers in areas of high development site density are more likely to be productive. The ecological setting and density of development sites should be incorporated in efforts to model and predict *Ae. aegypti* population levels. This understanding is vital in determining the feasibility of population control and the effort necessary to reduce vector populations below epidemic thresholds.

## Introduction

The primary mosquito vector of dengue viruses (DENV), *Aedes aegypti* (L.), is well adapted to living with people and in much of the world is predominantly found among human settlements.[1], [2] Most dengue illness similarly occurs in urban and peri-urban environments, where humans are the only vertebrate host. Immature *Ae. aegypti* develop in artificial and natural water-holding containers located in and around human habitations. Reducing or eliminating larval habitat has been advocated as an important component of sustainable vector control programs.[3], [4], [5] Although none are currently commercially available, vaccines effective against all four DENV serotypes are reaching the final stages of development.[6], [7] In the near future, a combined dengue prevention strategy involving vaccine deployment and vector population reduction may significantly reduce the global burden of dengue illness.[8], [9]

A key to successful and sustainable vector control for dengue, whether it is done alone or with a vaccine, is a fundamental understanding of *Ae. aegypti* ecology that would allow predictions on their abundance through space and time.[8] A primary determinant of adult mosquito population density concerns the types and number of containers in a given environment. Adult production is unevenly distributed across potential larval development sites. In most cases, a few key types of containers are responsible for a large proportion of the pupal, and thus adult, production.[10], [11], [12] Protective measures such as lids, larvicide, removal of discarded and unused containers or biological agents have reduced adult vector population density.[2], [13] Container capacity, water temperature, source of water, and container location, all of which can vary seasonally,[14], [15], [16] have been cited as important ecological factors affecting production of adult *Ae. aegypti*.[12], [17] A number of studies have also found that *Ae. aegypti* abundance is not homogeneous among households, with disproportionate numbers of immature and adult mosquitoes clustered in key premises.[18], [19], [20] A study of *Ae. aegypti* production in Amercan Samoa found that containers were more productive on average in houses with a large number of containers.[21] To this point, the relationship between productivity and the spatial distribution of containers has not been rigorously examined. That is, how does the density of nearby development sites affect overall adult *Ae. aegypti* production?

After emergence, female mosquitoes mate and begin taking blood meals. The first gonotrophic cycle is completed several days later when eggs are oviposited into available containers. Egg development and oviposition continues, often with multiple blood meals per cycle.[22], [23] Mark-release-recapture studies have found that most adult *Aedes aegypti* do not disperse more than 200 m, and that many are captured within the house they were released or neighboring houses.[23], [24], [25], [26] It has also been observed that females are less likely to disperse from houses with a large number of available ovisposition sites.[27] Given that most *Aedes aegypti* do not disperse very far, we would expect that containers in close proximity to other productive containers are more likely to be oviposition sites and more likely to receive a large number of eggs. We hypothesize that, holding other attributes constant, containers in areas of dense larval habitat will have a greater probability of being productive and a greater abundance of pupae than areas where suitable, wet containers are rare and thus have a spatially dispersed distribution.

In this study we chose to focus on pupal production. In most environments, identification and enumeration of *Ae. aegypti* pupae is feasible and pupal counts can be correlated with adult *Ae. aegypti* density. Epidemiologically, pupae per person has been proposed as a measure of entomological risk for DENV transmission;[10] i.e., entomologic thresholds have been estimated for the minimum pupal density required to support epidemic DENV transmission.[28]

We used a hurdle regression model to test the hypothesis that density of local larval habitat is positively associated with production of *Ae. aegypti* pupae populations. We use presence and abundance of pupae in water holding containers as dependent variables representing pupal production. Our model was fit using 4 years of field data from rural Kamphaeng Phet, Thailand and it predicts pupal productivity at the individual container level. We used a hierarchical regression framework to account for variation in productivity among container types. This allowed us to determine differential effects of ecological factors across container types and different container densities. Many of the surveys we carried out were repeated in the same villages semiannually. By including varying intercepts by survey we could account for spatial and temporal dependence within surveys and understand the effects of repeated sampling on *Ae. aegypti* populations. Our overall aim was to better understand adult *Ae. aegypti* production at the scale of individual containers and to interpret our results in the context of targeted larval control; i.e., treatment, protection, and/or removal of the most productive containers.

## Materials and Methods

### Study Area

**Figure 1:**
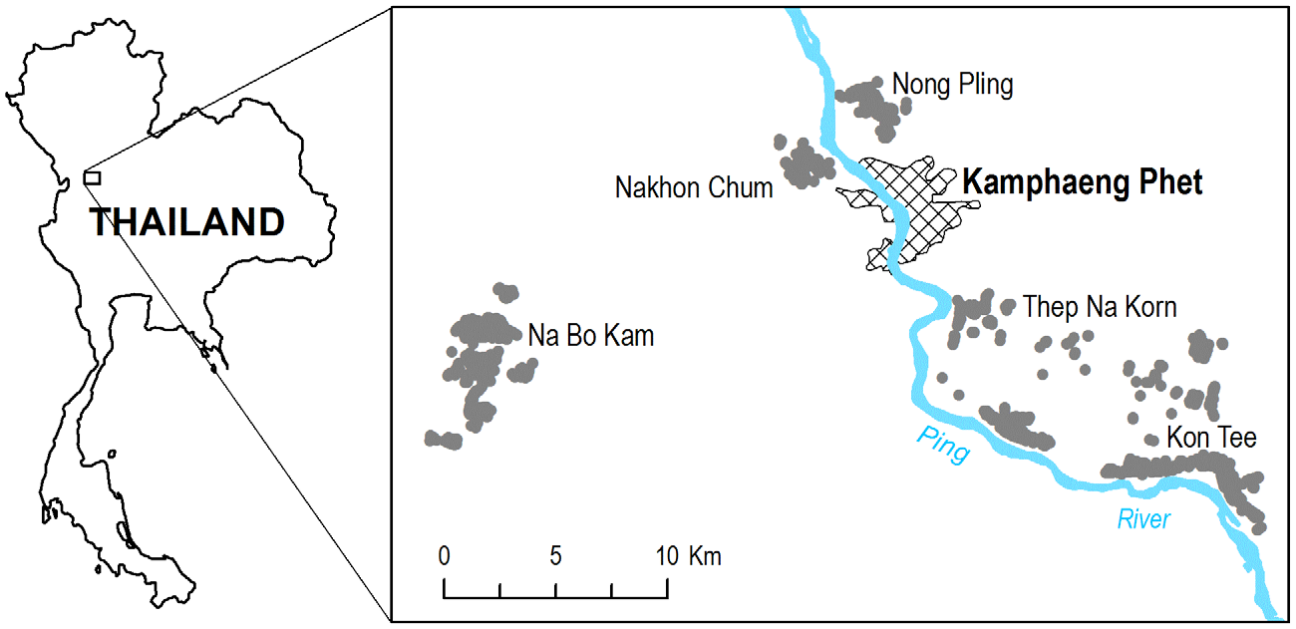
Study area. The gray areas indicate places where homes were mapped for survey in Kon Tee, Na Bo Kam, Thep Na Korn, Nakhon Chum, and Nong Pling sub districts. The cross hatching indicates the urbanized area of the city of Kamphaeng Phet, Thailand.

**Figure 2:**
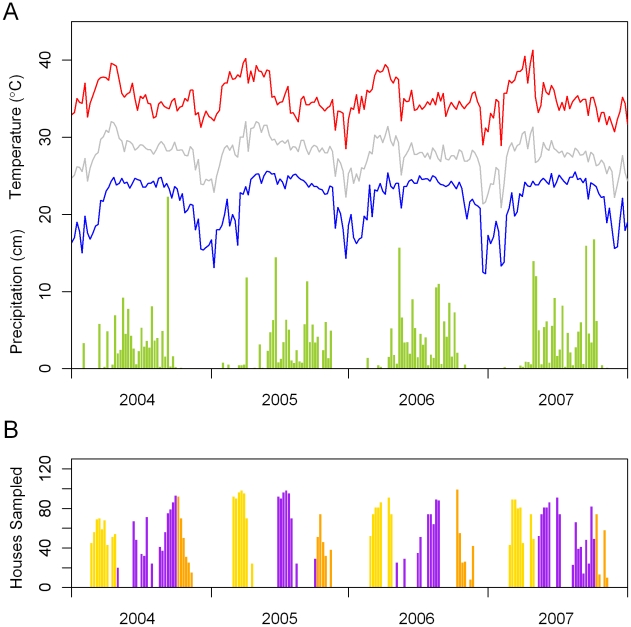
Meteorology and timing of samples. The lines in part A show the weekly maximum, minimum, and mean temperatures for the study area. The height of the bars indicates the weekly precipitation totals. Part B shows the number of households sampled per week during the study period. The yellow bars represent the early season samples, and the orange bars represent the late season samples. The purple bars indicate samples during the transmission season, primarily cluster surveys.

Our data came from pupal surveys conducted in Kamphaeng Phet Province, Thailand (Figure 1). Sampled households were part of an epidemiologic study that included more than 8,000 households within five sub-districts in the vicinity of the provincial capital.[14] People living in the study area experience symptomatic DENV infections annually and all four serotypes have been recovered from the region.[29], [30], [31] The climate is tropical with marked rainfall seasonality (Figure 2A).[32] The area has an active vector control program, which includes larvicide application and insecticide fumigation. In some cases, larvicides are distributed as a preventative control measure. Focal fumigation and larvicide application are also initiated by local public health authorities upon identification of dengue cases in their catchment area.

### Pupal Surveys

Pupal surveys were carried out from 2004 to 2007 (Figure 2B).[14] Approximately 6,400 household surveys were made in 2,088 unique households. Written consent was obtained from an adult resident of each household before surveys were conducted. The study protocol and consent forms were approved by the AFRIMS Scientific Review Committee and the ethical review committees of the U.S. Army Surgeon General, Thai MoPH, and University of California, Davis. The location of each household was captured using a global positioning system (GPS) receiver. All water-holding containers in each of these household plots were examined. For each container the following attributes were recorded: container type, container dimensions, water depth, temephos, an organophosphate larvicide, status (with/without), cover status (with/without), filling method (rain/manual), location (indoor/outdoor with some shade/full sun), and fish status (with/without). All pupae were collected from each container and reared to adults in our field laboratory. The sex and species of each emerging adult was determined and totals were associated with each container. A previous study in this region indicated that fumigation can lead to significant, although short lived, reductions in *Ae. aegypti* adult populations.[33] Containers in households that have been fumigated within the 2 months prior to the sampling date were removed from our analysis. In total, 84,840 out of 98,862 container samples were retained for this analysis.

Two types of sampling methods were employed: cross-sectional and cluster sampling. Each of the sampling methods was spatially exhaustive, in that all occupied household plots in study area were included. Cross-sectional sampling was performed in two sub-districts: Kon Tee (16°22′ N, 99°38′ E) and Na Bo Kam (16°24′ N, 99°22′ E).[14] Within each sub-district one village with high housing density and one village with low housing density were selected for survey. Each of the four villages was sampled twice per year with first survey at the end of the dry season (late March to early May) and the second at the end of the wet season (September through early November). The number of participating households varied based on occupancy and participation, with an average of 543 houses surveyed in each of the eight samples. Households were also sampled as part of a cluster sampling methodology.[31] For these surveys, households within 100 m and including the home of a child with overt dengue illness or a non-dengue febrile illness were examined. Symptomatic febrile illness was detected using a school based surveillance program. Cluster studies took place between June and November of each year. Some households in clusters were also participants in our biannual entomological survey described above. In addition to the Kon Tee and Na Bo Kam sub-districts, cluster samples were carried out in Thep Na Korn (16°24′ N, 99°32′ E), Nakhon Chum (16°29′ N, 99°30′ E), and Nong Pling (16°32′ N, 99°30′ E) sub-districts (See Figure 1).

### Variables

Our analyses focused on production of *Ae. aegypti* pupae. Our first dependent variable was whether or not there were any *Ae. aegypti* pupae in a container and is referred to as positive (POS). This variable is equal to one if at least one *Ae. aegypti* adult emerged from the pupae collected from a container and was otherwise equal to zero. Our second dependent variable was the total number of *Ae. aegypti* adults that emerged from pupae collected from a container. This variable is referred to as *Ae. aegypti* pupae (AEGPUP).

Containers were categorized to facilitate analysis. Container categories were based on five characteristics. The first concerned the type of container (jar, tank, tire, etc.). The remaining factors were temephos status, lid status, fill method, and location. Containers with fish were combined into one group. Classifications with fewer than 90 total containers were grouped into “Other.” A complete list of the 124 classifications used is provided in Table S1.

The PIPED variable indicates whether the container was located at a house with piped water or if water was drawn from a communal well. The samples were divided into three seasons that correspond approximately to the early season survey (EARLY), cluster surveys, and late wet season survey (LATE). The monsoon season cluster samples were our reference group. In some years cluster surveys continued into late fall. Those cluster surveys taking place in the 42^nd^ week of the year or later are classified as LATE to preserve temporal consistency. Timing and coding of the samples is shown in Figure 2B.

Density of larval habitats in the vicinity of a container is included in the model as a function of the Euclidean distance to nearby containers and the average productivity of container types. This measure can be thought of as a spatially weighted count of the expected number of pupae positive development sites in the vicinity of an individual container. Containers within the same household were given a spatial weight of one. This weight declines exponentially until containers at households beyond 50 m have a spatial weight near zero. The spatial weighting function was chosen to reflect the relatively short dispersal distances of *Ae. aegypti*.[24] Maximum range was also limited by the size and shape of sampling regions. The density of nearby larval habitats (DENS) was computed with the equation:
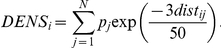



In this equation, *p_j_* is the proportion of all containers of the same classification as container *j* that are *Ae. aegypti* pupae positive (see Table S1). The variable *dist_ij_* is the distance in meters between the household where container *i* was sampled and the household where container *j* was sampled. The minimum, maximum, mean, and standard deviation of the observed DENS values are 0.026, 15, 3.6, and 2 respectively.

### Statistical Analysis

Container classifications significantly more likely to contain *Ae. aegypti* pupae than the remaining containers were determined using *Fisher's exact test*.[34], [35] For each test, a 2×2 contingency table was examined where the rows represent whether or not a container is observed with pupae. The columns of the table are the containers of the classification under evaluation and all other containers. A one-tailed test was conducted for each container classification with experiment wise *α = 0.05*. A Bonferroni correction was used to account for multiple testing, which yields a per comparison *α = 0.0004*.[36] The R software system was used to compute the statistics.[37]

A varying coefficients negative binomial hurdle model was estimated to examine effects of habitat density and season on *Ae. aegypti* pupal production.[38] Units of analysis were samples of water holding containers. Hurdle models are a type of discrete mixture model used for count data with excess zeros. Excess zeros results when a dependent variable contains more zeros than would be expected for a Poisson or negative binomial distribution. If the excess zeros are ignored parameter estimates and standard errors may be biased.[39] In this implementation, a logistic regression model was used to predict the probability that an observation crosses the hurdle (has a non-zero count). The non-zero counts were modeled as a truncated negative binomial distribution, where values were restricted to be greater than or equal to 1.

Dependent variables in the model were POS and AEGPUP. Independent variables were DENS, PIPED, EARLY, and LATE. Separate intercepts and slopes for each container classification were estimated. In the case of the negative binomial model, a shape parameter was also estimated for each container classification. The shape parameter is inversely proportional to the overdispersion or extra-Poisson variation. Additionally, an intercept that varied by sample was included. This second varying intercept was included in the model to account for spatial and temporal dependence not explained by the independent variables. The logistic regression model is
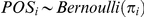


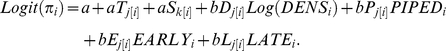



In these equations, *π_i_* is the probability that pupae are observed in container *i*. *a* is an overall intercept parameter. *aT_j_* and *aS_k_* are group-level intercepts for container classifications and surveys respectively. The parameters *bD_j_*, *bP_j_*, *bE_j_*, and *bL_j_* are group-level slopes estimated for each container classification.

The truncated negative binomial portion of the hurdle model is given as
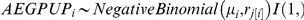


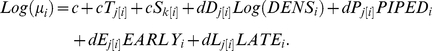

*µ_i_* is the expected value of the distribution and *r_j_* is the size parameter for each container classification. In this model, *c*, *cT_j_*, and *cS_k_* are the overall and group-level intercepts. The group-level slopes are *dD_j_*, *dP_j_*, *dE_j_*, and *dL_j_*. All four of the group-level intercept parameters were constrained to have an overall mean of zero; therefore, they represent deviations from the global intercept parameters. Non-informative prior probability distributions were assigned to each of the model parameters, indicating that no prior knowledge was incorporated into the parameter estimation.

The parameters were estimated using Bayesian Markov chain Monte Carlo (MCMC) methods available in the WINBUGS 1.4 software.[40] Convergence diagnostics and model evaluation were facilitated by the R2WinBugs package.[41] After a burn in period of 100,000 iterations, the posterior parameter distributions were sampled from the subsequent 20,000 iterations. Every 20^th^ iteration was retained, and estimates of the median and 95% credible interval were based on 1,000 samples. Convergence was confirmed by running multiple chains and examining the potential scale reduction factor.[42] The fit of the model was examined through the use of summary measures and stochastic simulation. The concordance index, used to measure the fit of the logistic regression model, is an estimate of the probability that the predictions and outcomes are concordant, and is equivalent to the area under the receiver operating characteristic (ROC) curve.[34], [43] The stochastic simulation procedure uses the fitted model parameters to create realizations that were compared to the observed data, and is a fundamental methodology for checking model fit.[44] The distributions of pupal counts for simulated datasets were examined at the individual container level and for aggregate pupae counts by survey.

## Results

### Container Productivity

The 22 key container classifications that were significantly more likely to contain pupae than the remaining containers are summarized in Table 1. Together, these classifications make up 36% of all samples, 76% of the pupae positive samples, and 79% of the *Ae. aegypti* pupae collected. Container classes most likely to be positive were ant traps and jars that were manually-filled and unprotected by temephos or a lid (Table 1). Together, jars, tanks, and ant traps made up a large majority of the productive containers. Eighty percent of the sampled containers were manually-filled, and these accounted for approximately the same percentage of total pupae. Containers located in full sun were less likely to be positive than corresponding container classifications located indoors or outdoors with some shade.

**Table 1:** The 22 key container classifications ordered by total *Ae. aegypti* pupae.

Type	Temephos	Lidded	Rain-Filled	Location	Samples	*Ae. agypti* Positive	Proportion Positive (*p_j_*)	*Ae. agypti* Pupae	Pupae per Container	Pupae per Positive Container
Jar	No	No	No	Outdoor	3408	947	0.278	6947	2.038	7.336
Tank	No	No	No	Indoor	1913	614	0.321	4535	2.371	7.386
Ant Trap	No	No	No	Indoor	2473	937	0.379	3309	1.338	3.531
Tank	No	No	No	Outdoor	1825	412	0.226	3131	1.716	7.600
Jar	No	No	No	Indoor	1142	428	0.375	2392	2.095	5.589
Jar	No	No	No	Full Sun	2284	319	0.140	2010	0.880	6.301
Jar	No	Yes	No	Indoor	3510	609	0.174	2005	0.571	3.292
Jar	No	Yes	No	Outdoor	3134	441	0.141	1568	0.500	3.556
Jar	No	No	Yes	Outdoor	1080	250	0.231	1460	1.352	5.840
Tire	No	No	Yes	Outdoor	821	243	0.296	1242	1.513	5.111
Tank	Yes	No	No	Indoor	1781	201	0.113	1221	0.686	6.075
Bucket	No	No	No	Indoor	1882	208	0.111	976	0.519	4.692
Tire	No	No	Yes	Full Sun	712	176	0.247	876	1.230	4.977
Tank	No	No	Yes	Outdoor	346	62	0.179	845	2.442	13.629
Jar	No	No	Yes	Full Sun	1171	163	0.139	725	0.619	4.448
Bucket	No	No	Yes	Outdoor	812	119	0.147	590	0.727	4.958
Ant Trap	No	No	No	Outdoor	475	175	0.368	573	1.206	3.274
Drum	No	No	No	Indoor	430	82	0.191	372	0.865	4.537
Cup	No	No	No	Indoor	411	88	0.214	338	0.822	3.841
Vase	No	No	No	Indoor	505	70	0.139	273	0.541	3.900
Drum	No	No	Yes	Outdoor	144	27	0.188	265	1.840	9.815
Tray	No	No	No	Indoor	108	30	0.278	175	1.620	5.833
Total					30367	6601	0.217	35828	1.180	5.428

### Model Fit

**Figure 3:**
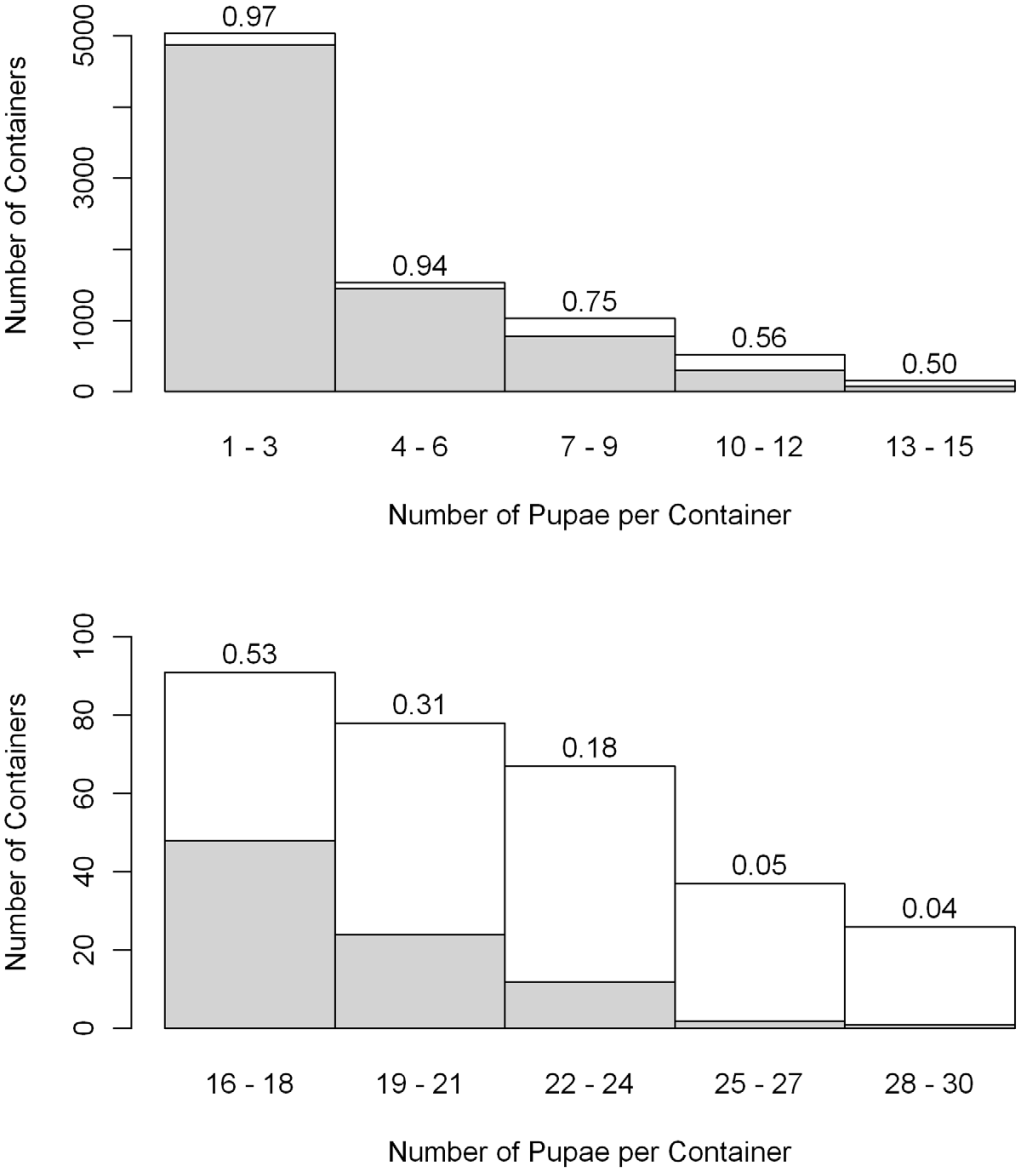
Container level model fit. The height of each bar indicates the total number of containers with observed pupae counts in each abundance category. The gray portion indicates the number of containers that the observed count of Ae. aegypti pupae fall between the 2.5 and 97.5 percentile of realizations generated using the estimated model and the proportions are given above each bar. The height of the white bars indicates the number of containers with pupal counts above the 95% simulation envelope.

The concordance index for the logistic portion of the hurdle model is 0.84, where perfect prediction of pupal presence would yield a value of 1.0 and random guessing or an intercept only model would yield a value of 0.5. One thousand realizations of pupal counts were generated using the estimated parameters of the entire hurdle model for each sampled container. Observed pupal counts were compared with the 2.5 and 97.5 percentiles of the realizations. Ninety-eight percent of the observed values were between the two percentile thresholds. All of the 76,137 observed zero counts were within the 95% simulation envelope, and 85% of the 8703 pupae positive containers were within the envelope. As pupal abundance increased container counts were less likely to fall within the envelope (Figure 3). More than 30 *Ae. aegypti* pupae were collected from 125 containers and all of those containers fell outside of the simulation envelope; nevertheless, most observed counts were not far above the simulation envelope. 52% of the observed counts above the envelope were within 3 pupae of the 97.5 percentile, and 99.8% of observed counts were less than the maximum simulated value (1000 realizations) for the container. At the container level, the overall model fit is adequate. The pupal counts for the super-producing containers, however, were underestimated.

**Figure 4:**
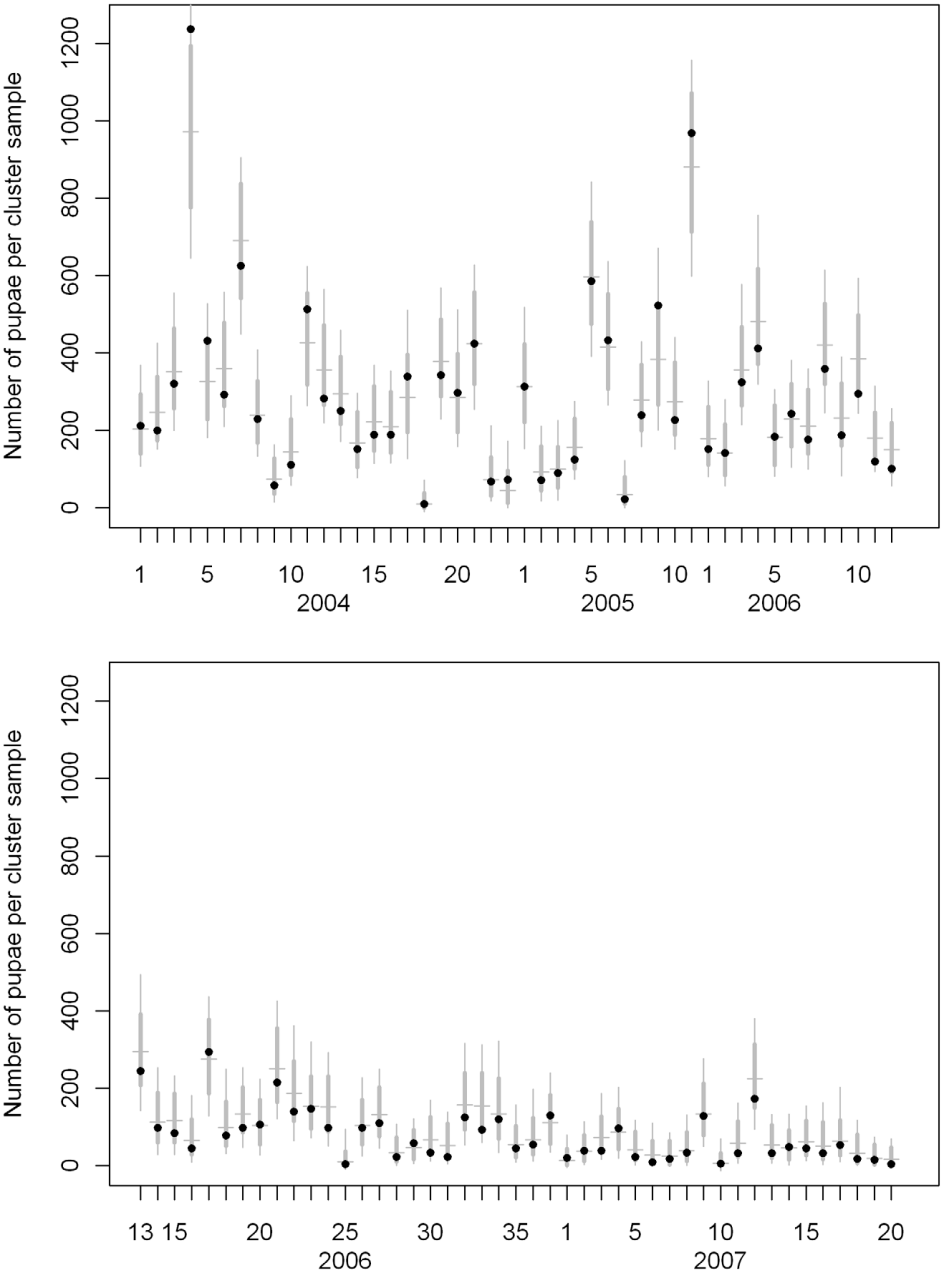
Cluster survey *Ae. aegypti* pupal population prediction. The black dots indicate the number of pupae collected during cluster surveys (2004–2007). The horizontal gray line indicates the median of 1000 simulation values. The thick gray vertical line indicates the central 95% of simulation values, and the thin gray vertical line indicates the range of all the simulations.

**Figure 5:**
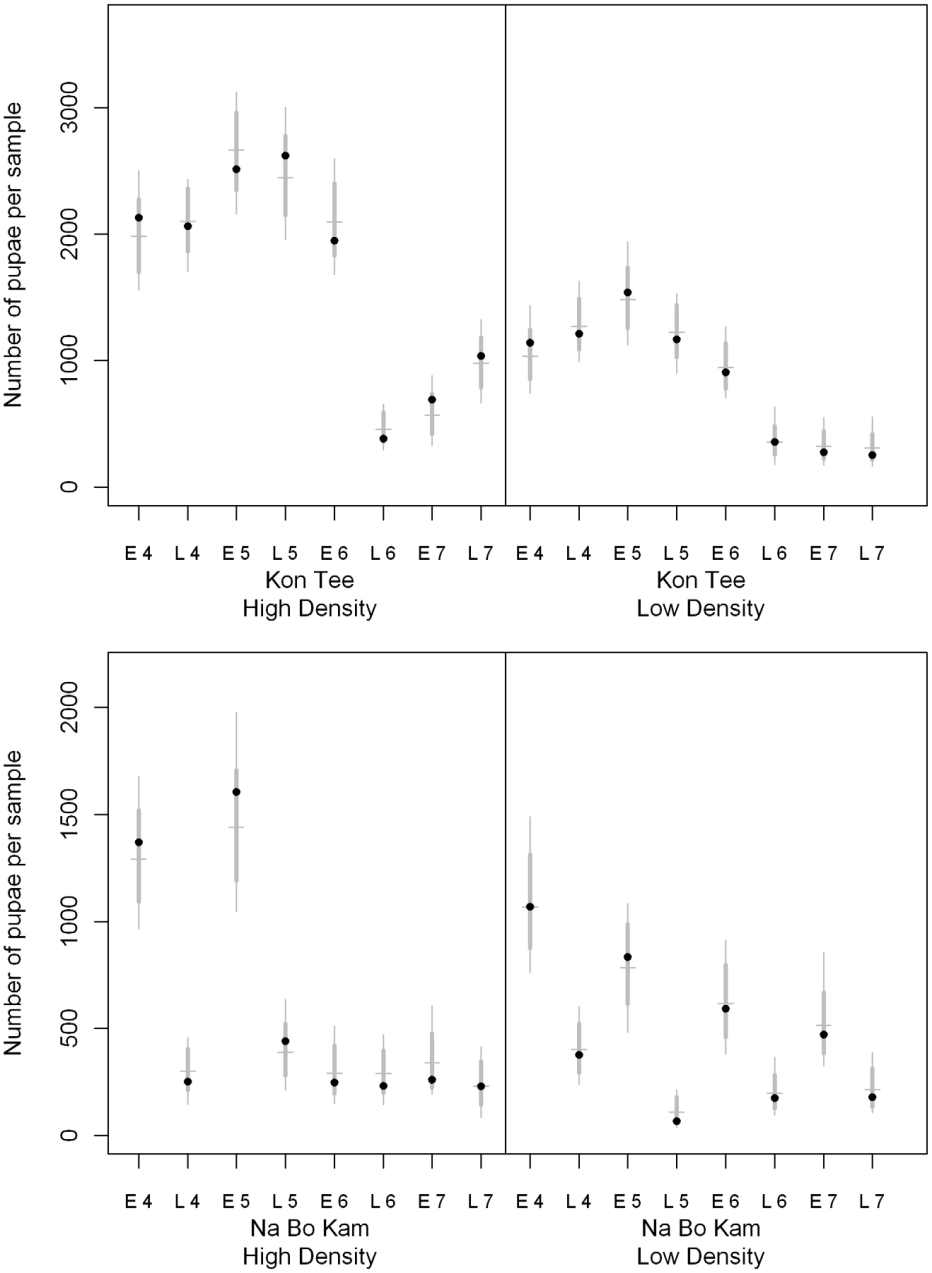
Cross sectional survey *Ae. aegypti* pupal population prediction. The black dots indicate the number of pupae collected during the repeated early and late season village surveys (2004–2007). The horizontal gray line indicates the median of 1000 simulation values. The thick gray vertical line indicates the central 95% of simulation values, and the thin gray vertical line indicates the range of all the simulations.

Simulation values were aggregated by sample to assess model fit over time and in different villages. Aggregated simulation values are shown for cluster surveys with at least 20 containers in Figure 4. The observed number of pupae per cluster survey was above the 95% simulation envelope in 3 out of 91 included cluster surveys. In each of the repeated samples the observed sum of pupae fell within the simulation envelope (Figure 5). These simulations indicate that observed aggregate pupal counts per sample are reproduced by the estimated model.

### Parameter Estimates

Table 2 shows the population parameter estimates. The median of the 1000 retained samples is given to indicate the center of the posterior parameter distribution, and the precision of each estimate is indicated by the 2.5 and 97.5 percentiles. The intercept coefficients presented in Table 2 are the average intercepts for the entire population and group-level intercepts represent deviations from these values. Remaining coefficients in Table 2 are the population parameters or the weighted average of the individual container classification slope parameters. The average slope for the DENS variable is positive for both portions of the model. These values indicate that as container density increases, the probability that pupae are found in individual containers and the expected abundance of pupae counts are higher. For the logistic model, the median population parameter estimate for the DENS variable is 0.47. This indicates that holding all else equal, a change in DENS from 1.6 (one standard deviation below the mean) to 5.6 (one standard deviation above the mean) leads to a change in the probability that a container is pupae positive from 0.053 to 0.080; a 51% increase in probability of pupae presence. For some container classifications the effect is much larger, with a predicted 100% or greater increase in probability of being pupae positive with the same change in container density. For the negative binomial portion of the model, the median population parameter estimate is 0.11. This parameter value indicates that a change in the DENS variable from 1.6 to 5.6 leads to a change in the expected number of pupae in positive containers from 3.9 to 4.2 (8% increase).

**Table 2:** Population level parameters estimates and their confidence intervals for the hurdle regression model.

	Logistic Model			Negative Binomial Model		
Parameter	Median	2.5%	97.5%	Median	2.5%	97.5%
Intercept	−2.7	−2.8	−2.5	1.4	1.3	1.5
DENS	0.47	0.34	0.58	0.11	0.04	0.18
PIPED	−0.20	−0.32	−0.07	0.01	−0.04	0.06
EARLY	−0.46	−0.62	−0.29	0.15	0.06	0.26
LATE	0.17	0.01	0.31	−0.11	−0.25	−0.04

Containers located in households with piped water had a lower probability of producing pupae, but there was not a significant effect on the pupae counts in positive containers. For the season variables EARLY and LATE, average effects are different for logistic and negative binomial models. In comparison to the cluster surveys during the monsoon season, containers sampled in the early part of the year were less likely to contain pupae, but the counts of positive containers are higher. The converse is true in the late season when the probability of observing pupae in containers was higher, but the pupae counts were lower on average.

**Figure 6:**
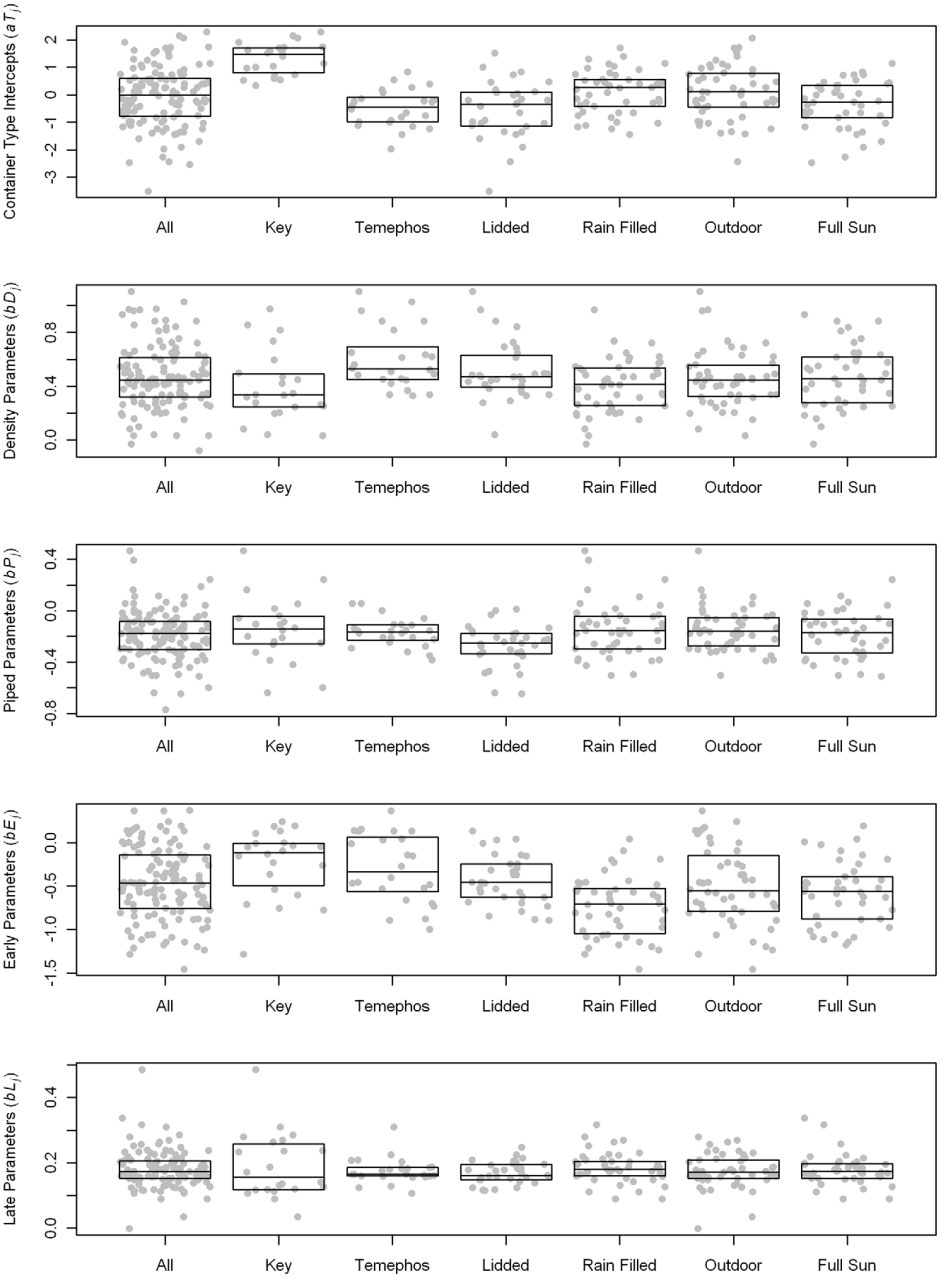
Container classification group parameters for the logistic portion of the hurdle regression model. The boxes represent the inter quartile range of the parameters, and the horizontal line within the box indicates the median value. The points representing individual parameter values are randomly displaced across the width of the box. The groups are not mutually exclusive. The center offset for each container classification is the same for the entire figure.

The variability among group-level parameters is shown in Table 3. For both models container classification intercepts show the most variation. Distributions of group-level parameters for the logistic model are shown in Figure 6. The variations in container type intercepts represent the relative differences in probability that containers contain pupae. As expected, key containers (Table 1) have mostly large positive intercepts indicating that the probability of being positive is relatively high. The temephos-treated and lidded containers have mostly negative intercepts indicating relatively low probabilities of being positive. The group-level density parameters indicate the relative effect of local development site density on container productivity. In the logistic model, several of the largest DENS variable slopes were for temephos-treated containers. The PIPED slope parameters indicate the effect of piped water being available in a household on container productivity in the household. The overall effect of having piped water is for lower productivity, but several rain-filled container classifications had positive slopes, indicating that they were on average more productive in households with piped water. The largest positive PIPED slope parameter (0.467) was for the key classification, buckets that were unprotected, rain-filled, and outdoors. The lowest PIPED slope parameter (−0.772) was for bottles that were unprotected, manually-filled, and indoors, indicating that these were less productive in houses with piped water. Rain-filled container classes have mostly low values for EARLY season parameter, indicating that these containers are less productive during the dryer part of the year. Manually-filled key classifications make up most of the large EARLY parameters, indicating that these containers remain productive during the dry season. Many of the temephos treated classifications also have large EARLY slope parameters. There was less variation among the LATE slope parameters. This lack of variation indicates that there were no large changes in relative productivity among container types between the cluster season and the late season.

**Table 3:** Group-level variability among model parameters.

		Logistic Model	Negative Binomial Model
Groups	Parameter	Standard Deviation	Standard Deviation
Container Type	Intercept	1.20	0.29
	DENS	0.41	0.16
	PIPED	0.34	0.07
	EARLY	0.59	0.19
	LATE	0.19	0.25
Sample	Intercept	0.71	0.12

**Figure 7:**
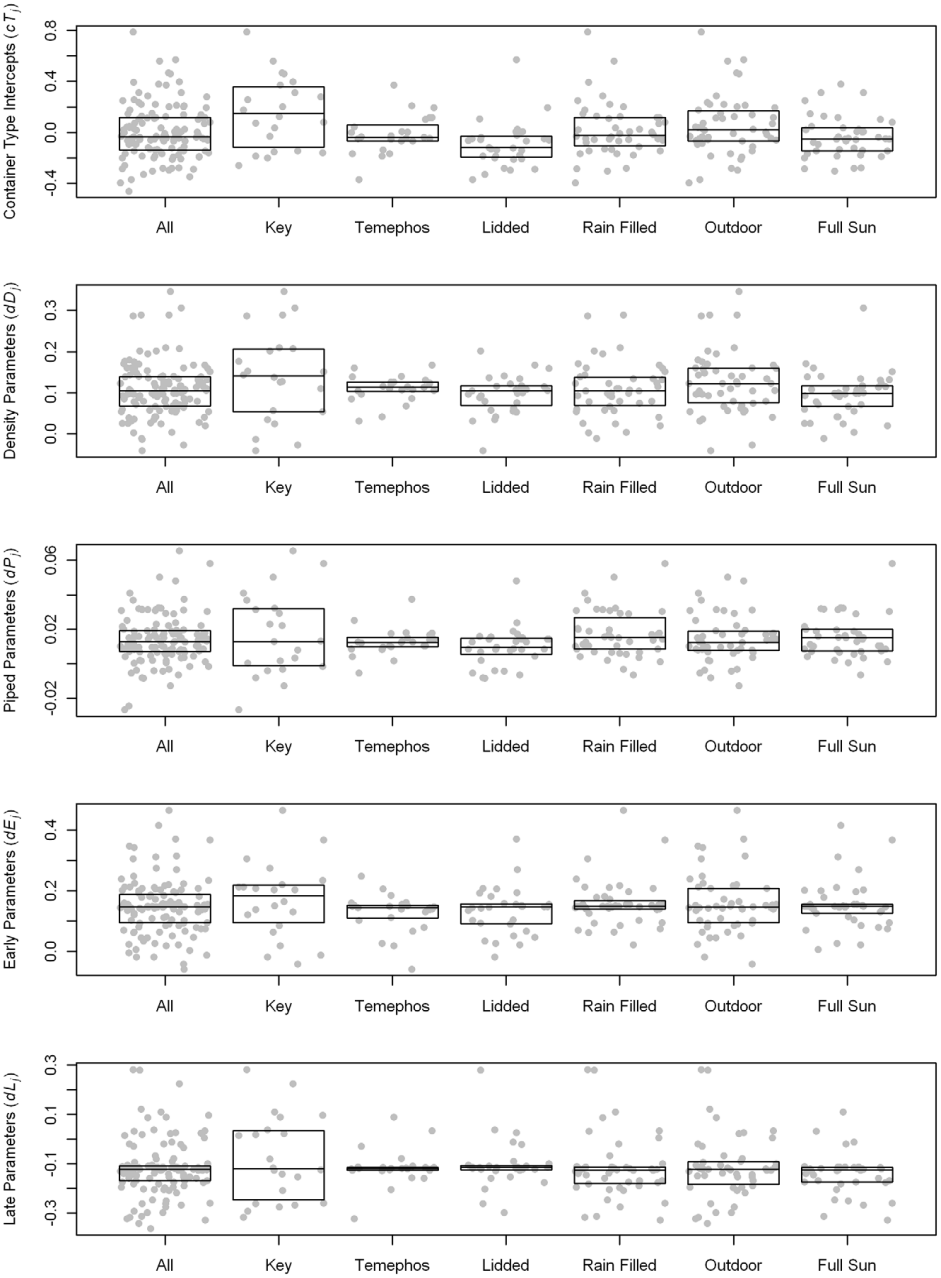
Container classification group parameters for the negative binomial portion of the hurdle regression model. The boxes represent the inter quartile range of the parameters, and the horizontal line within the box indicates the median value. The points representing individual parameter values are randomly displaced across the width of the box. The groups are not mutually exclusive. The center offset for each container classification is the same for the entire figure.

**Figure 8:**
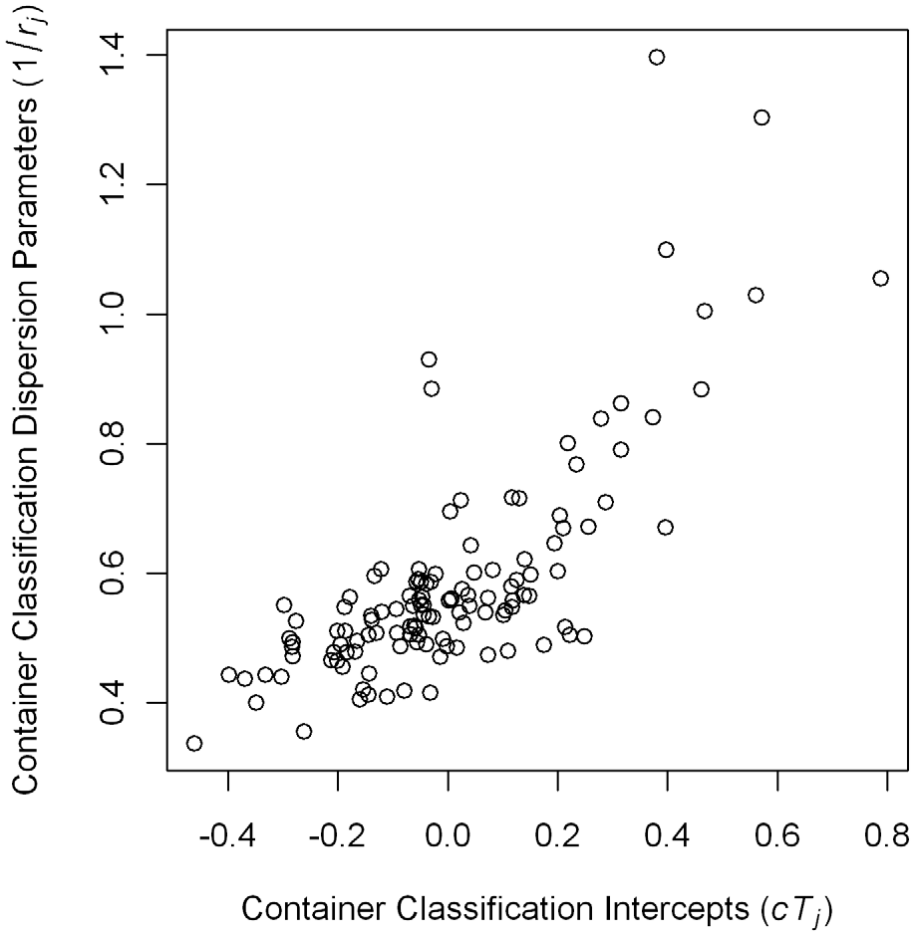
The relationship between container classification group intercepts and dispersion parameters. The container classification group intercepts and dispersion parameters for the negative binomial portion of the hurdle regression model.

The distributions of group-level parameters for the negative binomial model are shown in Figure 7. The number of protected containers that were pupae positive and, therefore, included in the negative binomial model were relatively small. Given the small numbers of observations in each group, parameters tended to shrink towards the overall mean. Container type intercepts indicated differences in mean pupae counts among positive containers. The largest intercept (0.788) was for unprotected tanks that were rain-filled and outdoors. Positive containers with lids had relatively low pupae counts. The relationship between container group intercepts and the dispersion parameter is shown in Figure 8. Container types with higher average counts had larger variance. The data points in the upper right quadrant of the plot are different classifications of tanks, drums, and jars. These large size containers had higher average pupal counts and were more likely than other types to have unusually large pupal counts.

**Figure 9:**
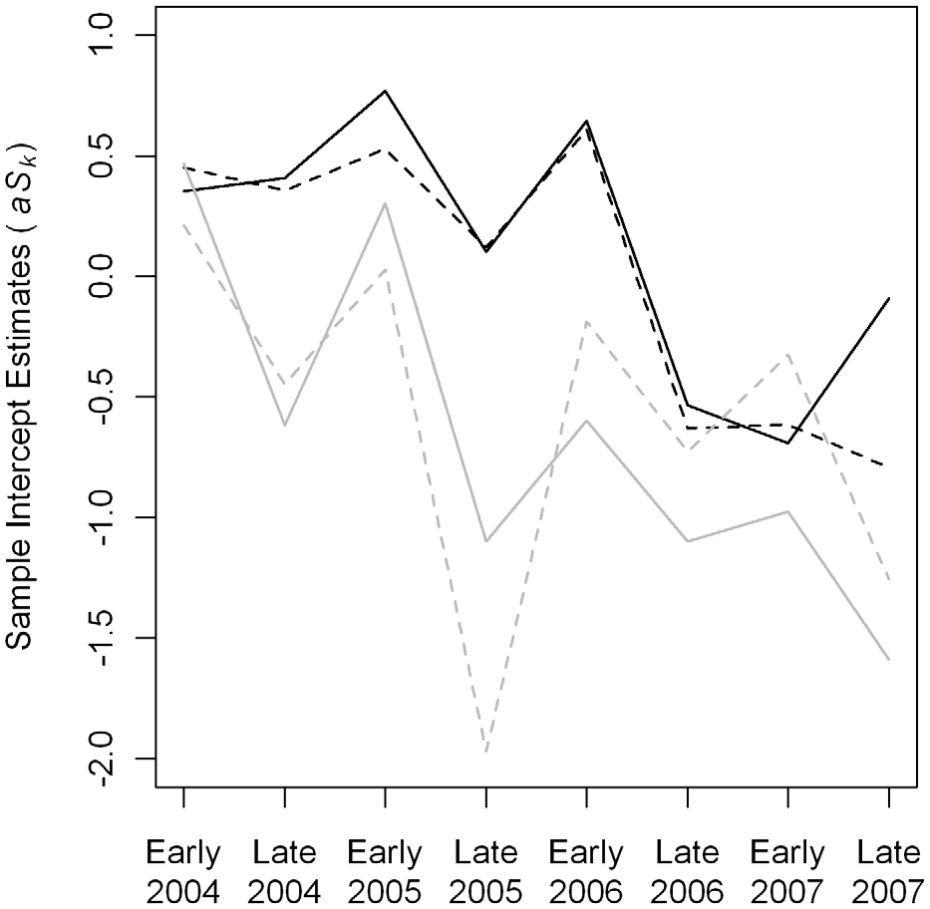
Posterior distribution medians for the logistic regression sample intercepts. The black lines are villages in the Kon Tee sub-district, and the gray lines are villages in the Na Bo Kam sub-district. Solid lines indicate the higher density villages, and dashed lines are the lower density villages.

Variation among sample level intercepts indicates that there is spatial and temporal variation in container productivity that is not explained by the variables included in the model. The overall trend is for lower productivity over the study period (Figure 9). In most years, parameter values were higher in the early than late sample. Intercept values for the Kon Tee sub-district were generally higher than those for Na Bo Kam sub-district. There did not appear to be large and systematic differences between intercept estimates for villages with a high density of houses and villages with a low-density of houses.

## Discussion

**Figure 10:**
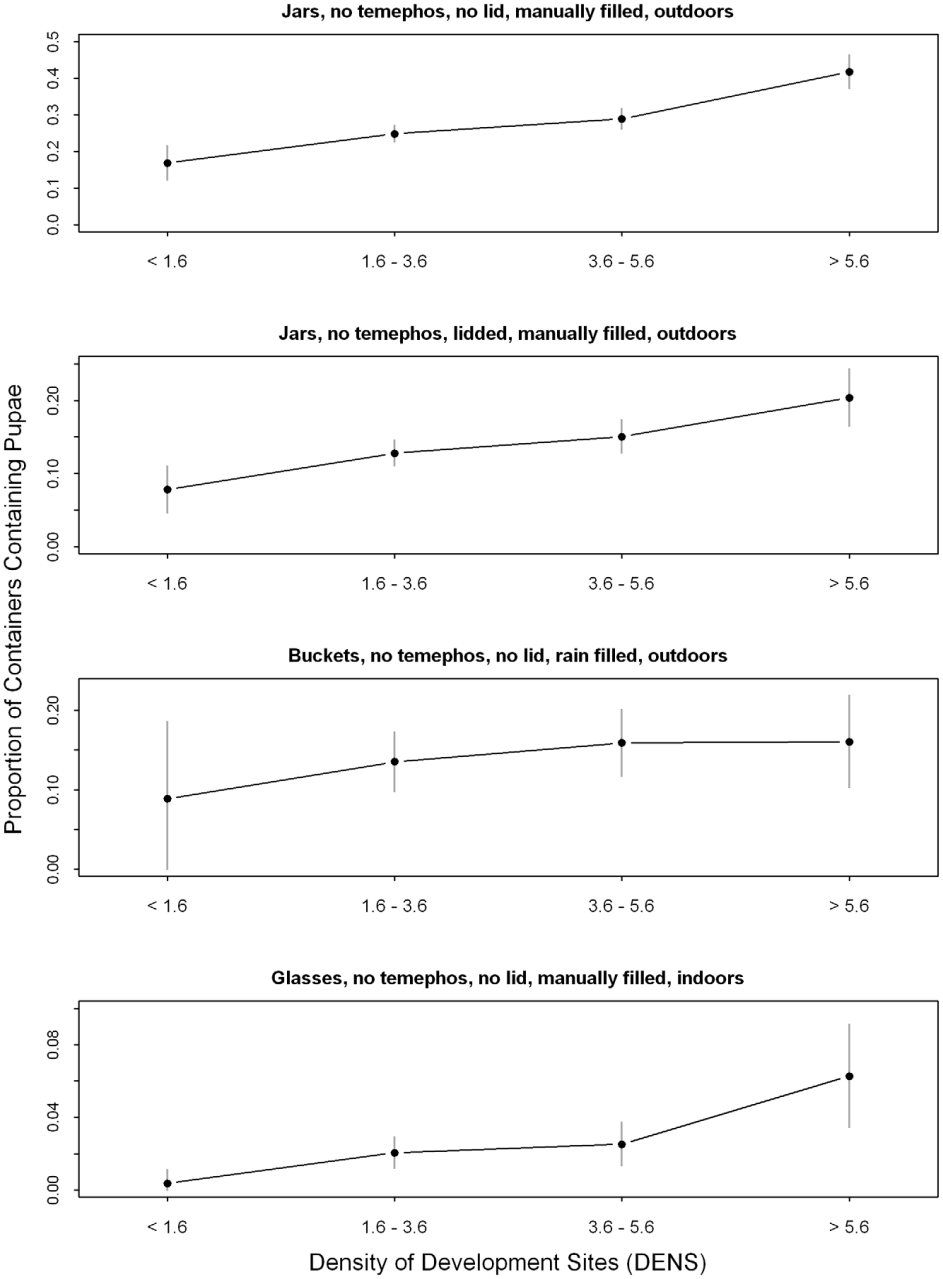
The relationship between productivity and development site density for four container classifications. The dots indicate the proportion of containers with Ae. aegypti pupae. The vertical bars represent the 95% confidence interval for the proportions. Note that the vertical scales are different for each container classification.

Our results support the hypothesis that containers in close proximity to other larval habitats will have a greater probability of being productive and a greater abundance of pupae than areas where larval habitats are spatially dispersed. Figure 10 shows differences in the proportion of containers that were pupae positive as density increased across four container classifications. These classifications were chosen to represent very productive containers, containers that were protected, rain-filled containers, and infrequently productive containers. This density relationship is consistent and statistically significant across different container types, seasons, and locations within the study area. The use of a varying coefficients model allowed us to incorporate container characteristics, including shade, location, lids, and application of larvicide, that were collected for each container. These results do not rule out the possibility that an unobserved factor is correlated with container density and is responsible for part or all of the effect. The density of larval habitats should be incorporated into future studies of container breeding mosquitoes and the results presented here should be confirmed in other locations.

The findings also support the concept of targeted larval control. Isolation of potential oviposition sites reduced the likelihood that they would contain pupae, and reduced the average abundance of pupae found in containers. Incorporating this density effect is crucial to properly estimating the effort required to lower vector population levels below an epidemic transmission threshold. Density had a large effect on the productivity of the protected container types, indicating that effectiveness of control measures will diminish as larval habitat density increases. Further field studies and ecological studies from other regions of the world will be useful in explicitly describing the spatial extent and biological mechanisms underpinning the relationship between larval development site density and container productivity. For example, we expect that manually-filled containers used to store water for domestic use will be especially important. They sustain population levels during extended dry seasons when rain-filled containers are empty and egg survival is challenged. Careful and exhaustive control during dry periods has the potential to improve overall vector population reduction efforts during other times of the year.

Although our results are consistent with targeted larval control, the operational challenge is that the strategy must be applied thoroughly in time and space. If areas of dense larval habitat are missed or if there are breaks in intervention at critical times, prospects for a successful outcome will be reduced. However, if it can be applied properly, which is not a trivial operational feat, our analyses indicate that removal of key development sites will result in decreased productivity from treated/removed containers as well as other containers that remain nearby.

The consistently productive container types in the Kamphaeng Phet area were primarily domestic water storage containers, such as jars and tanks followed by ant traps, buckets, tires, and smaller domestic containers. This corroborates results from previous studies in Kamphaeng Phet and other regions in Thailand.[4], [11], [14] Hurdle regression model estimates indicated that container classification accounted for the largest proportion of the variation in individual container productivity. Ecological variables such as habitat density, water source, seasonal variations, and neighborhood conditions during surveys played a significant role in container productivity.

Overall productivity of containers declined during the course of the study. Repeated sampling is a likely explanation for the cross-sectional surveys, but a similar decline was observed in areas without repeated sampling. During 2004 and 2005 DENV-4 was the primary circulating virus and most of the illnesses were not severe.[31] In the following seasons (2006 and 2007), there was an increased incidence and severity of dengue illness. Use of vector control was incorporated into our data selection and modeling process, but unspecified factors such as behavior changes may have accounted for some of the spatiotemporal variation in productivity between surveys. A knowledge, attitude, and practice (KAP) survey carried out in our study area reported regional variation in the proportion of households with knowledge of dengue and application of vector control methods that could also partially explain the presence of positive containers.[45] Longitudinal, integrated studies of epidemiological, entomological, and behavioral factors would be necessary to examine changes in practice corresponding to dengue burden and the resulting effect on vector populations.

Our regression model provides reasonable estimates of container productivity at the individual container and sample level. The model did not, however, adequately predict the number of pupae emerging from a small number super-producing containers. The two-part hurdle model effectively accounted for the large number of zero pupal counts. We used a truncated negative binomial distribution to characterize the number of pupae per positive container with a separate dispersion parameter for each container classification. Larger containers had the highest means and dispersion parameters. The increased flexibility of this approach performs better than a single negative binomial model at characterizing the distribution of larval counts, but this rarely resulted in pupae estimates greater than 30 for an individual container. Dispersion parameter estimates were influenced by the large proportion of the pupae positive containers that produced a small number of pupae, and did not fully characterize the observed distribution of pupae per container. Underestimates for survey-wide pupae counts were also due to super-producing containers, but their influence and frequency declined over the course of the study period.

A closer examination of the containers mostly likely to produce large numbers of pupae illustrates the difficulties in effectively modeling the distribution of pupal counts in super producing containers. Eighty-one of the 125 container samples (65%) with more than 30 pupae were collected in untreated, un-lidded, and manually-filled jars and tanks. For these large containers, the distribution of positive containers and pupae counts are given in Table 4. Even among these large containers, only 3% of the positive containers had more than 30 pupae. The maximum number of emerging pupae collected from a single container was 298. The proportion of containers that are pupae positive, the proportion that contain more than 30 pupae and the maximum pupae counts increase with development site density. Even in the high density category, however, 88% of the positive containers have 10 or fewer pupae and 57% of the positive containers have 4 or fewer pupae. This large number of small pupal counts has a large weight in parameter estimation relative to the few very large pupae counts.

**Table 4:** The relationship between *Ae. aegypti* productivity and development site density for unprotected water storage jars and tanks.

Jars and Tanks that are Untreated, Unlidded, and Manually-filled	Low Density(DENS <1.6)	Medium-Low Density(1.6< DENS <3.6)	Medium-High Density(3.6< DENS <5.6)	High Density(DENS >5.6)	Total
Total	892	5335	3363	1465	11055
Pupae Positive	126(0.141)	1130(0.212)	892(0.265)	528(0.360)	2676(0.242)
Number of samples with 1 pupae	30(0.034)[0.238]	292(0.055)[0.258]	224(0.067)[0.251]	140(0.096)[0.265]	686(0.062)[0.256]
Number of positive samples with 4 or fewer pupae	76(0.085)[0.603]	660(0.124)[0.584]	539(0.160)[0.604]	302(0.206)[0.572]	1577(0.143)[0.589]
Number of positive samples with 10 or fewer pupae	117(0.131)[0.929]	994(0.186)[0.880]	794(0.236)[0.890]	465(0.317)[0.881]	2370(0.214)[0.886]
Number of samples with greater than 30 pupae	1(0.001)[0.008]	28(0.005)[0.025]	31(0.009)[0.035]	21(0.014)[0.040]	81(0.007)[0.030]
Maximum observed pupae count	52	125	290	298	298

Values in parentheses indicate proportions of the total containers in each category. Values in brackets indicate the proportion of positive containers.

Super-producing containers tended to be relatively rare events, and may not be representative of the overall distribution of the standing crop of pupae. More importantly, the significance of super-producing containers in DENV transmission is not well documented. Further analysis is needed to examine the relationship between pupae production in general and the few very productive containers, especially with regard to the number of adults captured in nearby houses. Several field studies have implicated food limitation and larval competition as the primary regulating factors for *Aedes aegypti* larval development.[17], [46], [47] The frequency and pattern of water filling and removal may also play a role in the timing of pupal counts. These internal, ecological container characteristics were not captured in the current study, but should be incorporated along with local container density in ecological studies of *Ae. aegypti* production. Future modeling efforts should be designed to examine the likelihood of super-producing containers and better parameterize the distribution of pupal production in these habitats.

Piped water in a household had an overall negative effect on individual container productivity, but the classification level parameters varied considerably. The protective effect of piped water was strongest among manually-filled containers. A number of container classifications were more likely to produce pupae at households with piped water, primarily outdoor, rain-filled containers. Differences in water management related to household water source appear to have differential effects on container productivity. These results suggest that changes in water infrastructure may lead to changes in the relative productivity of different container types.

Population level parameters for the EARLY and LATE variables had opposite signs in the logistic and negative binomial models. The difference was driven by the relative productivity of manual and rain-filled containers. At the beginning of the wet season (EARLY), manually-filled containers were the primary producers. Most manually-filled containers were relatively large jars and tanks that when positive had higher pupae counts. During the wet season, rain-filled containers were more likely to be productive, but on average they produced fewer pupae. At the end of the wet season (LATE), almost all containers were again more likely to be productive, which further lowered overall productivity per container. For our logistic model, early season parameter estimates for larvicide treated containers (i.e., temephos) were on average high. This was likely due to the time elapsed between treatment during the previous transmission season and mosquito sampling almost one year later. Temephos packages were observed in the containers, but we suspect that their effectiveness had waned.

An improved understanding of the environmental determinants of container productivity is an important part of adaptive vector control efforts.[48] Studies are currently underway in tropical regions of the world to test the effect of container removal on *Ae. aegypti* populations.[49] Incorporating development site density into those efforts along with research on the ecology within larval development sites will lead to a more cohesive understanding of *Ae. aegypti* population dynamics and contribute to the design of increasingly effective vector interventions.

## Supporting Information

Table S1 A complete listing of container classifications used in the study. The list is arranged alphabetically by type, followed by containers with fish and other classifications.
(0.28 MB DOC)
