# Supplementary material for: Ecological Modeling of Aedes aegypti (L.) Pupal Production in Rural Kamphaeng Phet, Thailand
Source: PLoS Negl Trop Dis. 2011 Jan 18;5(1):e940. doi: 10.1371/journal.pntd.0000940 (PMC3022520; doi:10.1371/journal.pntd.0000940)
Supplement: Table S1 — A complete listing of container classifications used in the study. The list is arranged alphabetically by type, followed by containers with fish and other classifications. (0.28 MB DOC) [file pntd.0000940.s001.doc]

| Type | Teme-phos | Lidded | Rain Filled | Location | Samples | *Ae. aegypti* Positive | Proportion Positive | *Ae. aegypti* Pupae | Pupae per Container |
| --- | --- | --- | --- | --- | --- | --- | --- | --- | --- |
| Ant Trap | Yes | No | No | Indoor | 1134 | 8 | 0.007 | 34 | 0.030 |
| Ant Trap | Yes | No | No | Outdoor | 224 | 1 | 0.004 | 1 | 0.004 |
| Ant Trap | No | No | No | Indoor | 2473 | 937 | 0.379 | 3309 | 1.338 |
| Ant Trap | No | No | No | Outdoor | 475 | 175 | 0.368 | 573 | 1.206 |
| Bottle | No | Yes | No | Indoor | 3274 | 1 | 0.000 | 2 | 0.001 |
| Bottle | No | Yes | No | Outdoor | 1203 | 4 | 0.003 | 5 | 0.004 |
| Bottle | No | Yes | No | Full Sun | 177 | 0 | 0.000 | 0 | 0.000 |
| Bottle | No | No | No | Indoor | 487 | 28 | 0.057 | 111 | 0.228 |
| Bottle | No | No | No | Outdoor | 725 | 35 | 0.048 | 139 | 0.192 |
| Bottle | No | No | No | Full Sun | 415 | 3 | 0.007 | 7 | 0.017 |
| Bottle | No | No | Yes | Outdoor | 306 | 33 | 0.108 | 186 | 0.608 |
| Bottle | No | No | Yes | Full Sun | 455 | 31 | 0.068 | 109 | 0.240 |
| Bucket | Yes | No | No | Indoor | 105 | 3 | 0.029 | 14 | 0.133 |
| Bucket | No | Yes | No | Indoor | 2644 | 47 | 0.018 | 127 | 0.048 |
| Bucket | No | Yes | No | Outdoor | 1637 | 20 | 0.012 | 56 | 0.034 |
| Bucket | No | Yes | No | Full Sun | 135 | 2 | 0.015 | 3 | 0.022 |
| Bucket | No | No | No | Indoor | 1882 | 208 | 0.111 | 976 | 0.519 |
| Bucket | No | No | No | Outdoor | 2682 | 209 | 0.078 | 1094 | 0.408 |
| Bucket | No | No | No | Full Sun | 937 | 41 | 0.044 | 141 | 0.150 |
| Bucket | No | No | Yes | Outdoor | 812 | 119 | 0.147 | 590 | 0.727 |
| Bucket | No | No | Yes | Full Sun | 571 | 59 | 0.103 | 237 | 0.415 |
| Can | No | No | No | Indoor | 105 | 18 | 0.171 | 58 | 0.552 |
| Can | No | No | No | Outdoor | 114 | 15 | 0.132 | 80 | 0.702 |
| Can | No | No | Yes | Outdoor | 178 | 20 | 0.112 | 47 | 0.264 |
| Can | No | No | Yes | Full Sun | 195 | 15 | 0.077 | 36 | 0.185 |
| Chicken Feeder | No | No | No | Outdoor | 108 | 7 | 0.065 | 23 | 0.213 |
| Coconut | No | No | Yes | Outdoor | 215 | 8 | 0.037 | 35 | 0.163 |
| Coconut | No | No | Yes | Full Sun | 291 | 12 | 0.041 | 26 | 0.089 |
| Cover | No | No | Yes | Outdoor | 174 | 19 | 0.109 | 78 | 0.448 |
| Cover | No | No | Yes | Full Sun | 149 | 3 | 0.020 | 3 | 0.020 |
| Cup | No | No | No | Indoor | 411 | 88 | 0.214 | 338 | 0.822 |
| Cup | No | No | No | Outdoor | 206 | 14 | 0.068 | 69 | 0.335 |
| Cup | No | No | Yes | Outdoor | 169 | 11 | 0.065 | 47 | 0.278 |
| Cup | No | No | Yes | Full Sun | 258 | 4 | 0.016 | 30 | 0.116 |
| Drum | Yes | No | No | Indoor | 104 | 3 | 0.029 | 13 | 0.125 |
| Drum | No | Yes | No | Indoor | 306 | 8 | 0.026 | 21 | 0.069 |
| Drum | No | Yes | No | Outdoor | 240 | 10 | 0.042 | 25 | 0.104 |
| Drum | No | No | No | Indoor | 430 | 82 | 0.191 | 372 | 0.865 |
| Drum | No | No | No | Outdoor | 398 | 55 | 0.138 | 321 | 0.807 |
| Drum | No | No | No | Full Sun | 138 | 12 | 0.087 | 95 | 0.688 |
| Drum | No | No | Yes | Outdoor | 144 | 27 | 0.188 | 265 | 1.840 |
| Drum | No | No | Yes | Full Sun | 101 | 15 | 0.149 | 43 | 0.426 |
| Glass | No | No | No | Indoor | 2354 | 59 | 0.025 | 133 | 0.056 |
| Glass | No | No | No | Outdoor | 510 | 5 | 0.010 | 32 | 0.063 |
| Glass | No | No | No | Full Sun | 952 | 4 | 0.004 | 15 | 0.016 |
| Glass | No | No | Yes | Outdoor | 174 | 13 | 0.075 | 48 | 0.276 |
| Glass | No | No | Yes | Full Sun | 480 | 13 | 0.027 | 54 | 0.113 |
| Jar | Yes | Yes | No | Indoor | 105 | 2 | 0.019 | 3 | 0.029 |
| Jar | Yes | Yes | No | Outdoor | 591 | 16 | 0.027 | 37 | 0.063 |
| Jar | Yes | Yes | No | Full Sun | 129 | 11 | 0.085 | 33 | 0.256 |
| Jar | Yes | No | No | Indoor | 412 | 41 | 0.100 | 211 | 0.512 |
| Jar | Yes | No | No | Outdoor | 1845 | 91 | 0.049 | 399 | 0.216 |
| Jar | Yes | No | No | Full Sun | 360 | 17 | 0.047 | 68 | 0.189 |
| Jar | Yes | No | Yes | Outdoor | 142 | 5 | 0.035 | 13 | 0.092 |
| Jar | No | Yes | No | Indoor | 3510 | 609 | 0.174 | 2005 | 0.571 |
| Jar | No | Yes | No | Outdoor | 3134 | 441 | 0.141 | 1568 | 0.500 |
| Jar | No | Yes | No | Full Sun | 686 | 82 | 0.120 | 309 | 0.450 |
| Jar | No | Yes | Yes | Outdoor | 2929 | 63 | 0.022 | 270 | 0.092 |
| Jar | No | Yes | Yes | Full Sun | 1261 | 21 | 0.017 | 67 | 0.053 |
| Jar | No | No | No | Indoor | 1142 | 428 | 0.375 | 2392 | 2.095 |
| Jar | No | No | No | Outdoor | 3408 | 947 | 0.278 | 6947 | 2.038 |
| Jar | No | No | No | Full Sun | 2284 | 319 | 0.140 | 2010 | 0.880 |
| Jar | No | No | Yes | Outdoor | 1080 | 250 | 0.231 | 1460 | 1.352 |
| Jar | No | No | Yes | Full Sun | 1171 | 163 | 0.139 | 725 | 0.619 |
| Pan | No | Yes | No | Indoor | 158 | 1 | 0.006 | 9 | 0.057 |
| Pan | No | Yes | No | Outdoor | 111 | 1 | 0.009 | 1 | 0.009 |
| Pan | No | No | No | Indoor | 577 | 9 | 0.016 | 58 | 0.101 |
| Pan | No | No | No | Outdoor | 628 | 10 | 0.016 | 74 | 0.118 |
| Pan | No | No | No | Full Sun | 280 | 7 | 0.025 | 13 | 0.046 |
| Pan | No | No | Yes | Outdoor | 101 | 16 | 0.158 | 78 | 0.772 |
| Plate | No | No | Yes | Full Sun | 99 | 1 | 0.010 | 4 | 0.040 |
| Pot | No | No | Yes | Full Sun | 128 | 10 | 0.078 | 22 | 0.172 |
| Sheet | No | No | Yes | Outdoor | 205 | 19 | 0.093 | 134 | 0.654 |
| Sheet | No | No | Yes | Full Sun | 278 | 9 | 0.032 | 46 | 0.165 |
| Tank | Yes | No | No | Indoor | 1781 | 201 | 0.113 | 1221 | 0.686 |
| Tank | Yes | No | No | Outdoor | 2037 | 113 | 0.055 | 502 | 0.246 |
| Tank | No | Yes | No | Indoor | 237 | 5 | 0.021 | 23 | 0.097 |
| Tank | No | Yes | No | Outdoor | 292 | 11 | 0.038 | 214 | 0.733 |
| Tank | No | No | No | Indoor | 1913 | 614 | 0.321 | 4535 | 2.371 |
| Tank | No | No | No | Outdoor | 1825 | 412 | 0.226 | 3131 | 1.716 |
| Tank | No | No | No | Full Sun | 483 | 37 | 0.077 | 366 | 0.758 |
| Tank | No | No | Yes | Outdoor | 346 | 62 | 0.179 | 845 | 2.442 |
| Tank | No | No | Yes | Full Sun | 275 | 29 | 0.105 | 119 | 0.433 |
| Tile | No | No | No | Indoor | 1521 | 65 | 0.043 | 327 | 0.215 |
| Tile | No | No | No | Outdoor | 2395 | 64 | 0.027 | 345 | 0.144 |
| Tile | No | No | No | Full Sun | 975 | 5 | 0.005 | 24 | 0.025 |
| Tile | No | No | Yes | Outdoor | 428 | 34 | 0.079 | 138 | 0.322 |
| Tile | No | No | Yes | Full Sun | 286 | 10 | 0.035 | 39 | 0.136 |
| Tire | No | No | No | Outdoor | 206 | 30 | 0.146 | 146 | 0.709 |
| Tire | No | No | No | Full Sun | 117 | 11 | 0.094 | 71 | 0.607 |
| Tire | No | No | Yes | Outdoor | 821 | 243 | 0.296 | 1242 | 1.513 |
| Tire | No | No | Yes | Full Sun | 712 | 176 | 0.247 | 876 | 1.230 |
| Tray | No | No | No | Indoor | 108 | 30 | 0.278 | 175 | 1.620 |
| Tray | No | No | No | Outdoor | 146 | 9 | 0.062 | 26 | 0.178 |
| Tray | No | No | Yes | Outdoor | 92 | 8 | 0.087 | 30 | 0.326 |
| Tray | No | No | Yes | Full Sun | 107 | 10 | 0.093 | 39 | 0.364 |
| Vase | No | No | No | Indoor | 505 | 70 | 0.139 | 273 | 0.541 |
| Vase | No | No | No | Outdoor | 206 | 13 | 0.063 | 46 | 0.223 |
| Vase | No | No | No | Full Sun | 885 | 20 | 0.023 | 75 | 0.085 |
| Vase | No | No | Yes | Full Sun | 529 | 20 | 0.038 | 66 | 0.125 |
| Fish | No | No | No | Full Sun | 3997 | 21 | 0.005 | 48 | 0.012 |
| Other | Yes | Yes | No | Indoor | 31 | 2 | 0.065 | 7 | 0.226 |
| Other | Yes | Yes | No | Outdoor | 46 | 2 | 0.043 | 21 | 0.457 |
| Other | Yes | Yes | No | Full Sun | 3 | 0 | 0.000 | 0 | 0.000 |
| Other | Yes | Yes | Yes | Outdoor | 79 | 2 | 0.025 | 2 | 0.025 |
| Other | Yes | Yes | Yes | Full Sun | 24 | 0 | 0.000 | 0 | 0.000 |
| Other | Yes | No | No | Indoor | 160 | 2 | 0.013 | 7 | 0.044 |
| Other | Yes | No | No | Outdoor | 222 | 5 | 0.023 | 17 | 0.077 |
| Other | Yes | No | No | Full Sun | 125 | 1 | 0.008 | 4 | 0.032 |
| Other | Yes | No | Yes | Indoor | 3 | 1 | 0.333 | 10 | 3.333 |
| Other | Yes | No | Yes | Outdoor | 76 | 3 | 0.039 | 18 | 0.237 |
| Other | Yes | No | Yes | Full Sun | 122 | 7 | 0.057 | 19 | 0.156 |
| Other | No | Yes | No | Indoor | 134 | 7 | 0.052 | 20 | 0.149 |
| Other | No | Yes | No | Outdoor | 55 | 2 | 0.036 | 5 | 0.091 |
| Other | No | Yes | No | Full Sun | 121 | 7 | 0.058 | 39 | 0.322 |
| Other | No | Yes | Yes | Indoor | 28 | 4 | 0.143 | 9 | 0.321 |
| Other | No | Yes | Yes | Outdoor | 86 | 9 | 0.105 | 28 | 0.326 |
| Other | No | Yes | Yes | Full Sun | 49 | 2 | 0.041 | 3 | 0.061 |
| Other | No | No | No | Indoor | 103 | 16 | 0.155 | 56 | 0.544 |
| Other | No | No | No | Outdoor | 337 | 40 | 0.119 | 204 | 0.605 |
| Other | No | No | No | Full Sun | 281 | 16 | 0.057 | 55 | 0.196 |
| Other | No | No | Yes | Indoor | 229 | 62 | 0.271 | 377 | 1.646 |
| Other | No | No | Yes | Outdoor | 474 | 54 | 0.114 | 195 | 0.411 |
| Other | No | No | Yes | Full Sun | 416 | 43 | 0.103 | 212 | 0.510 |
| Total |  |  |  |  | 84840 | 8703 | 0.103 | 45227 | 0.533 |
